# Metabolic liver function in humans measured by 2-^18^F-fluoro-2-deoxy-D-galactose PET/CT–reproducibility and clinical potential

**DOI:** 10.1186/s13550-017-0320-1

**Published:** 2017-08-29

**Authors:** Kirstine P. Bak-Fredslund, Peter Lykke Eriksen, Ole L. Munk, Gerda E. Villadsen, Susanne Keiding, Michael Sørensen

**Affiliations:** 1 Department of Nuclear Medicine & PET Centre, Aarhus University Hospital, Aarhus, Denmark; 2 Department of Hepatology and Gastroenterology, Aarhus University Hospital, Noerrebrogade 44, DK-8000 Aarhus, Denmark

**Keywords:** Metabolic liver function, Galactose, Positron emission tomography, Molecular imaging, Remnant liver function

## Abstract

**Background:**

PET/CT with the radioactively labelled galactose analogue 2-^18^F-fluoro-2-deoxy-D-galactose (^18^F-FDGal) can be used to quantify the hepatic metabolic function and visualise regional metabolic heterogeneity. We determined the day-to-day variation in humans with and without liver disease. Furthermore, we examined whether the standardised uptake value (SUV) of ^18^F-FDGal from static scans can substitute the hepatic systemic clearance of ^18^F-FDGal (*K*_met_, mL blood/min/mL liver tissue/) quantified from dynamic scans as measure of metabolic function.

Four patients with cirrhosis and six healthy subjects underwent two ^18^F-FDGal PET/CT scans within a median interval of 15 days for determination of day-to-day variation. The correlation between *K*_met_ and SUV was examined using scan data and measured arterial blood concentrations of ^18^F-FDGal (blood samples) from 14 subjects from previous studies. Regional and whole-liver values of *K*_met_ and SUV along with total metabolic liver volume and total metabolic liver function (total SUV, average SUV multiplied by total metabolic liver volume) were calculated.

**Results:**

No significant day-to-day differences were found for *K*_met_ or SUV. SUV had higher intraclass correlation coefficients than *K*_met_ (0.92–0.97 vs. 0.49–0.78). The relationship between *K*_met_ and SUV was linear. Total metabolic liver volume had non-significant day-to-day variation (median difference 50 mL liver tissue; *P* = 0.6). Mean total SUV in healthy subjects was 23,840 (95% CI, 21,609; 26,070), significantly higher than in the patients (*P* < 0.001).

**Conclusions:**

The reproducibility of ^18^F-FDGal PET/CT was good and SUV can substitute *K*_met_ for quantification of hepatic metabolic function. Total SUV of ^18^F-FDGal is a promising tool for quantification of metabolic liver function in pre-treatment evaluation of individual patients.

## Background

Non-invasive tests for evaluation of severity and prognosis of liver disease are major research areas in hepatology [1]. Imaging-based methods include ^99m^Tc-mebrofenin scintigraphy with SPECT [2], Gd-EOB-DTPA-enhanced MRI [3], and 2-^18^F-fluoro-2-deoxy-D-galactose (^18^F-FDGal) PET/CT [4]. An important finding from these methods is that the liver tissue becomes functionally heterogeneous in patients with parenchymal liver disease [2–5]. Functional heterogeneity is important when planning surgery or other local treatment modalities of liver tumours [6–8]. Removal of a small part of the anatomical liver volume in a patient with cirrhosis may reduce the liver function relatively more than expected from the CT volume, which may lead to liver failure [6].

Galactose is metabolised by cytosolic galactokinase in the hepatocytes and is used in the established galactose elimination capacity (GEC) test as a measure of the total metabolic liver function [9]. ^18^F-FDGal is a positron-labelled galactose analogue which is also metabolised by galactokinase, and ^18^F-FDGal PET/CT can be used to generate 3-dimensional images of the hepatic systemic clearance of ^18^F-FDGal (*K*_met_, mL blood/min/mL liver tissue), which correlates to the maximum hepatic removal rate of ^18^F-FDGal, i.e. a local GEC. ^18^F-F DGal PET/CT has been validated as a functional measure in pigs [10], healthy human subjects [11], and patients with cirrhosis [4].

For a method to be clinically applicable, it must be reproducible and relatively simple to perform and read. The aims of the present study were to determine the day-to-day variation of ^18^F-FDGal PET measurements and to test whether the standardised uptake value (SUV) of ^18^F-FDGal can be used as a substitute for *K*_met_ for quantification of regional and whole-liver metabolic function.

## Methods

### Human subjects

The day-to-day variations in *K*_met_ and SUV were analysed in six healthy subjects (five male, one female) and four patients with cirrhosis (one male, three female) who were prospectively included. Median age was 60 years for healthy subjects (range, 42–71 years) and 58 years for patients with cirrhosis (47–65 years). Median weight was 83 kg for healthy subjects (70–96 kg) and 55 kg for patients with cirrhosis (51–77 kg). Patients were recruited at the outpatient clinic at the Department of Hepatology and Gastroenterology, Aarhus University Hospital. Criteria for inclusion of the patients were biopsy-confirmed cirrhosis and stable disease as judged by biochemistry and clinical presentation. Patient characteristics are shown in Table 1. All patients were Child-Pugh class A [12]. Healthy subjects with no current signs or history of liver disease were included after responding to an advertisement on a Danish website dedicated to the recruitment of subjects for scientific experiments; all healthy subjects had normal blood biochemistry. The study was approved by The Central Denmark Region Committees on Health Research Ethics and conducted in accordance with the 1975 Declaration of Helsinki. Written informed consent was obtained from all subjects. No complications to the procedures were observed.

**Table 1:** Patient characteristics

ID	Sex/age (year)	Aetiology	ALT (U/L plasma)	Bilirubin (μmol/L plasma)	ALP (U/L plasma)	PP	Albumin (g/L plasma)	Platelets (10^9^/L whole blood)	Child-Pugh class
1	M/47	AIH	39	15	79	0.65	NA	134	A^a^
2	F/56	AIH/PBC overlap syndrome	71	35	291	0.67	32	74	A
3	F/59	Cryptogenic	40	16	77	0.51	40	85	A
4	F/65	PBC	20	16	125	0.76	32	140	A

*F* female, *M* male, *NA* not available, *ALT* alanine aminotransferase (normal < 70 U/L plasma for males < 45 U/L plasma for females); bilirubin (normal < 25 μmol/L plasma), *ALP* alkaline phosphatase (normal < 105 U/L plasma), *PP* coagulation factors II,VII, and X (normal > 0.6); albumin (normal > 36 g/L plasma); platelets (normal > 145 10^9^/L whole blood; Child-Pugh class, patients were scored according to the Child-Pugh classification [12], *AIH* autoimmune hepatitis, *PBC* primary biliary cholangitis

^a^CP score calculated assuming that albumin > 36g/L plasma

The correlation between SUV and *K*_met_ was analysed using raw data from 14 subjects from two previous publications [4, 11] in which the arterial concentrations of ^18^F-FDGal (kBq/mL blood) were measured in blood samples collected during the PET scans. We used data from experiments without simultaneous galactose infusion from six healthy subjects (all male) [11], mean age 60 years (55–65 years), mean weight 88 kg (73–92 kg), and eight patients with cirrhosis (seven male, one female) [4], mean age 62 years (43–66 years), mean weight 80 kg (62–93 kg); aetiologies were alcohol (*n* = 6), cryptogenic cirrhosis (*n* = 1), and mixed alcohol/HCV (*n* = 1). All healthy subjects had normal blood biochemistry. For the patients, median albumin was 37 g/L plasma (33–43 g/L plasma; normal > 36 g/L plasma), median bilirubin was 12 μmol/L plasma (4–20 μmol/L plasma; normal < 25 μmol/L plasma), median alanine aminotransferase was 26 U/L plasma (13–121 U/L plasma; normal < 70 U/L plasma), and median alkaline phosphatase was 96 U/L plasma (80–155 U/L plasma; normal < 105 U/L plasma); three patients were Child-Pugh class A and five were class B [4].

For all 12 patients with cirrhosis, the diagnosis was based on either biopsy or clinical work-up including ultrasound sonography and blood biochemistry according to current standard clinical practice.

### ^18^F-FDGal PET/CT

All 24 subjects were scanned in supine position using the same 64-slice Siemens Biograph TruePoint PET/CT camera (Siemens AG, Erlangen, Germany). The subjects fasted for at least 4 h before the scans but were allowed to drink water. For the day-to-day variation study, each subject underwent two dynamic ^18^F-FDGal PET/CT scans with a median interval of 15 days (7–53 days).

First, a topogram of the abdomen was performed for optimal positioning of the liver within the 21.6 cm transaxial field-of-view of the scanner followed by a low-dose CT scan (50 effective mAs with CAREDose4D, 120 kV, pitch 0.8, slice thickness of 5 mm) which was used for attenuation correction of PET data and anatomical co-registration with the PET images. A bolus of 100 MBq ^18^F-FDGal (96–113 MBq), produced at our radiochemistry facilities at the PET Centre, Aarhus University Hospital [13], was injected intravenously at the beginning of a dynamic PET scan of the liver (list mode acquisition). Scan time was 20 min for the subjects in the day-to-day variation study. For the analysis of a correlation between SUV and *K*_met_ [4, 11], only the initial 20 min were used for the present purpose.

The PET data were corrected for radioactive decay back to start of the scan and reconstructed without resolution modelling (6 iterations, 21 subsets and 2 mm Gaussian filter) and with resolution modelling (4 iterations, 21 subsets and 2 mm Gaussian filter) and with both 336 and 168 matrices. This yielded four sets of 3-dimensional images of the tissue radioactivity concentration (kBq/mL) for each scan.

The hepatic systemic clearance of ^18^F-FDGal (*K*_met_) was calculated voxel-by-voxel by applying the Gjedde-Patlak model of irreversible trapping of ^18^F-FDGal to data from 6 to 20 min after the ^18^F-FDGal injection using the time course of arterial tracer concentration as input [4, 10, 11]. An image-derived arterial input extracted from scan data using a volume of interest (VOI) placed in the abdominal aorta [14] (median volume 2.18 mL blood; range, 3.4–4.2 mL blood) was used in the day-to-day study and arterial tracer concentrations measured in blood samples were used in the analysis of a correlation between SUV and *K*_met_.

The standardised uptake value (SUV, unit less) was calculated as the mean tissue radioactivity concentration (kBq/mL) recorded by the PET camera 10 to 20 min after the ^18^F-FDGal injection divided by the injected dose per gram of body weight (kBq/g) and using an average tissue density of 1 g/mL tissue.

Regional values of *K*_met_ and SUV were acquired from a region of interest (ROI) drawn in the right lobe of the liver using the co-registered PET/CT images. The ROI was copied to at least six adjacent slices and summed into a liver VOI. The liver VOI was placed at least 1 cm from the edge of the liver and major blood vessels. In each subject, the same liver VOI was used for all four reconstructions, both for the *K*_met_ and SUV images.

The total functional liver volume (milliliter liver tissue) was calculated using a VOI encircling the whole liver using the iso-contour tool with a threshold of 6 kBq/mL tissue (approximately twice the radioactivity concentration in the abdominal aorta 15–20 min after tracer injection). The whole-liver VOIs were adjusted manually for any non-liver tissue (e.g. right kidney). The total metabolic liver function (total SUV) was calculated as the total functional liver volume multiplied by the average SUV in this volume.

PMOD software version 3.605 (PMOD Technologies Ltd., Switzerland) was used for all image analyses.

### Statistical analysis

Measurements from the day-to-day study were tested for normality using the Shapiro-Wilk test and compared using Student’s paired *t* test or the signed Rank test as appropriate. Day-to-day variation and the impact of reconstruction algorithm and matrix size were investigated by one-way ANOVA analysis (using ranks when Shapiro-Wilk test of normality failed). Correlations between measurements were evaluated by Pearson’s product-moment correlation coefficient (*r*). For linear regression analysis, the equations are listed with standard error of the estimate (± SE) and *R* ^2^, the coefficient of determination adjusted for the number of variables. Reliability of measurements was evaluated by intraclass correlation coefficient (ICC). A *P* value less than 0.05 was considered to indicate statistical significance. SigmaPlot for Windows (Version 11.0 build 11.2.0.5) and Stata version 14.2 (StataCorp, College Station, TX) were used for statistical calculations.

## Results

### Day-to-day variation and impact of reconstruction algorithm

**Fig. 1:**
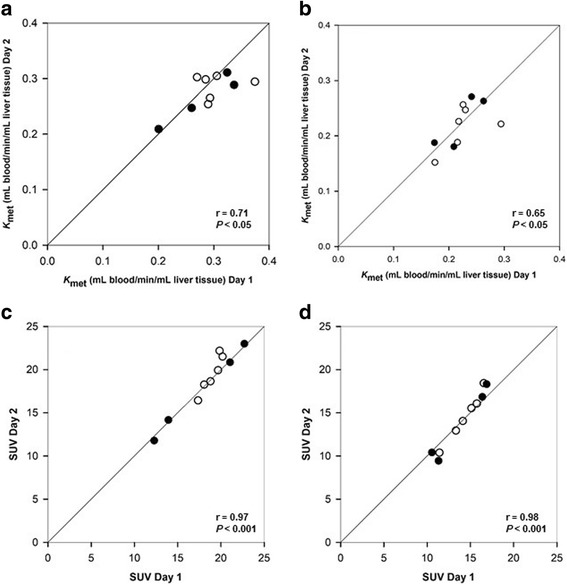
Correlations between *K*_met_ and SUV of ^18^F-FDGal for paired measurements in patients with cirrhosis (black circle) and healthy subjects (white circle). Regional VOI values (**a**, **c**) and whole-liver VOI values (**b**, **d**) for the reconstruction algorithm with resolution modelling and a 168 matrix are shown with lines of identity

There were no statistically significant differences between the day-to-day measurements of *K*_met_ or SUV for any of the four reconstruction algorithms; this applied to both the regional liver VOI and whole-liver VOI values (Fig. 1). The ICC for the four reconstruction methods ranged from 0.49 to 0.78 for *K*_met_ and from 0.92 to 0.97 for SUV. These results show that the estimation of SUV was more reliable and with lower measurement error than *K*_met_.

The impact of reconstruction algorithms on *K*_met_ in the day-to-day study showed significant differences in both regional and whole-liver values for the 336 matrix reconstructions when comparing with and without resolution modelling (regional *P* = 0.005 and whole-liver *P* = 0.006) whereas there were no significant differences for the 168 matrix reconstructions. There was no significant impact of the reconstruction algorithm on the SUV estimates for any of the four reconstruction methods or for regional or whole-liver values. When comparing individual *K*_met_ values calculated with measured arterial input (in the study of the correlation between SUV and *K*_met_), there were no differences between the reconstruction methods (regional *P* = 0.76 and whole-liver *P* = 0.65). These findings indicate that the image-derived input is sensitive to the reconstruction algorithm with measurement errors propagating into the estimation of *K*_met_.

### SUV as a substitute for *K*_met_

**Fig. 2:**
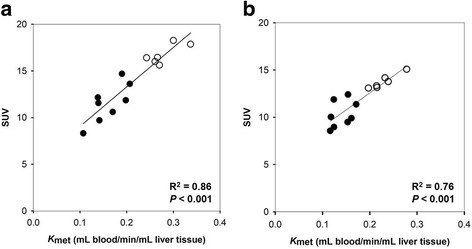
Correlations between regional values of SUV and *K*_met_ (**a**) and between whole-liver values of SUV and *K*_met_ (**b**) for ^18^F-FDGal PET/CT in patients with cirrhosis (black circle) and healthy subjects (white circle). Results from reconstruction algorithm with resolution modelling and a 168 matrix are shown with fitted linear regression lines

Due to the uncertainty in *K*_met_ measurements when using an image-derived input, the relation between SUV and *K*_met_ was examined using data from 14 subjects with arterial blood sampling [4, 11]. Linear regression for the regional liver VOI was SUV = 4.8 (SE ± 1.1) + 42.3 (SE ± 4.7) *K*_met_, *R* ^2^ = 0.86 (Fig. 2a), and for the whole-liver VOI, it was SUV = 5.4 (SE ± 1.0) + 35.9 (SE ± 5.5) *K*_met_, *R* ^2^ = 0.76 (Fig. 2b). The linear correlations between SUV and *K*_met_ demonstrate that SUV can substitute *K*_met_ as a measure of the metabolic liver function both regionally and for the whole liver. We therefore proceeded with SUV in the following calculations of the total functional liver volume and the total metabolic liver function.

### Total functional liver volume and total metabolic liver function

**Fig. 3:**
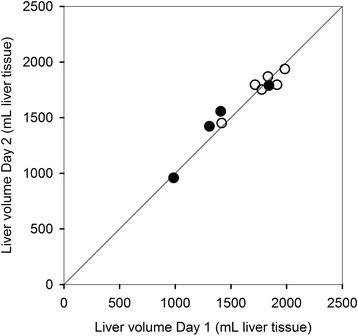
Paired measurements of total functional liver volume in patients with cirrhosis (black circle) and healthy subjects (white circle) with line of identity

On day 1, the mean total functional liver volume was 1621 mL liver tissue (985–1984 mL liver tissue), and on day 2, it was 1632 mL liver tissue (958–1936 mL liver tissue) (*P* = 0.60) (Fig. 3). The median day-to-day difference was 50 mL liver tissue (− 149–119 mL liver tissue).

Mean total SUV for the healthy subjects from both study cohorts (*n* = 11) was 23,840 (95% CI, 21,609; 26,070) with no significant difference between the four reconstruction algorithms (*P* = 0.3). One healthy subject was excluded from this analysis as the total functional liver volume was overestimated due to rigorous respiratory movements that resulted in a halo of recorded ^18^F-FDGal above the diaphragm. Total SUV was significantly higher in the healthy subjects than in the patients (*n* = 12) (*P* < 0.001).

## Discussion

The main finding of the present study is that SUV of ^18^F-FDGal can be used as a substitute for *K*_met_ of ^18^F-FDGal for quantification of regional hepatic metabolic function which simplifies the ^18^F-FDGal PET/CT method significantly. In addition, the day-to-day variation was low which is important for the clinical application of the method.

^18^F-FDGal PET/CT quantifies the cytosolic metabolic function in hepatocytes in terms of galactokinase capacity whereas methods such as ^99m^Tc-mebrofenin SPECT and Gd-EOB-DTPA MRI assess the uptake and excretory function of the hepatocytes and not metabolism [15]. The transport of ^18^F-FDGal into the hepatocyte is facilitated by glucose transporters while both ^99m^Tc-mebrofenin and Gd-EOB-DTPA are taken up by organic anion-transporting polypeptides and excreted by multidrug resistance protein 2 [15]. As a consequence of shared pathways of transport through the hepatocyte, hyperbilirubinemia may affect uptake and excretion of both ^99m^Tc-mebrofenin and Gd-EOB-DTPA, an issue that is not relevant for ^18^F-FDGal PET although we have only limited experiences with patients with severe hyperbilirubinemia.

*K*_met_ of ^18^F-FDGal is a validated measure of the hepatic metabolic function, viz. in vivo galactokinase capacity for hepatocellular phosphorylation of ^18^F-FDGal [4, 11], whereas SUV is derived from the PET-measured radioactivity concentration in liver tissue which includes not only metabolised ^18^F-FDGal in the hepatocytes but also any un-metabolised ^18^F- FDGal in blood and hepatocytes. Because the amount of un-metabolised ^18^F-FDGal with time becomes negligible compared to the amount of ^18^F-FDGal-1-phosphate [4, 11], we chose the time interval 10 to 20 min after tracer injection for the calculation of SUV. Moreover, generation of images of *K*_met_ requires an input of ^18^F-FDGal to the liver which can either be obtained from arterial blood sampling [4, 10, 11], which is discomforting for the patient, or by using an image-derived method [14]. The image-derived method is however sensitive to subtle movements of the subject during the scan, which can cause the VOI to be displaced outside of the aorta, and, as we show here, also depends on which reconstruction algorithm is applied to data. The latter is in agreement with the findings of a significant impact of reconstruction algorithms on radioactivity concentration measurements in sub-centimetre VOIs in phantoms [16].

In order to validate the present findings, we determined the linear correlation between *K*_met_ and SUV for the whole liver in a third cohort of nine patients with hepatocellular carcinoma, who were examined with arterial blood sampling [17]. In this cohort, the correlation was SUV = 4.2 (SE ± 1.9) + 38.9 (SE ± 13) *K*_met_ (R^2^ = 0.50) which was quite close to that in the present study.

**Fig. 4:**
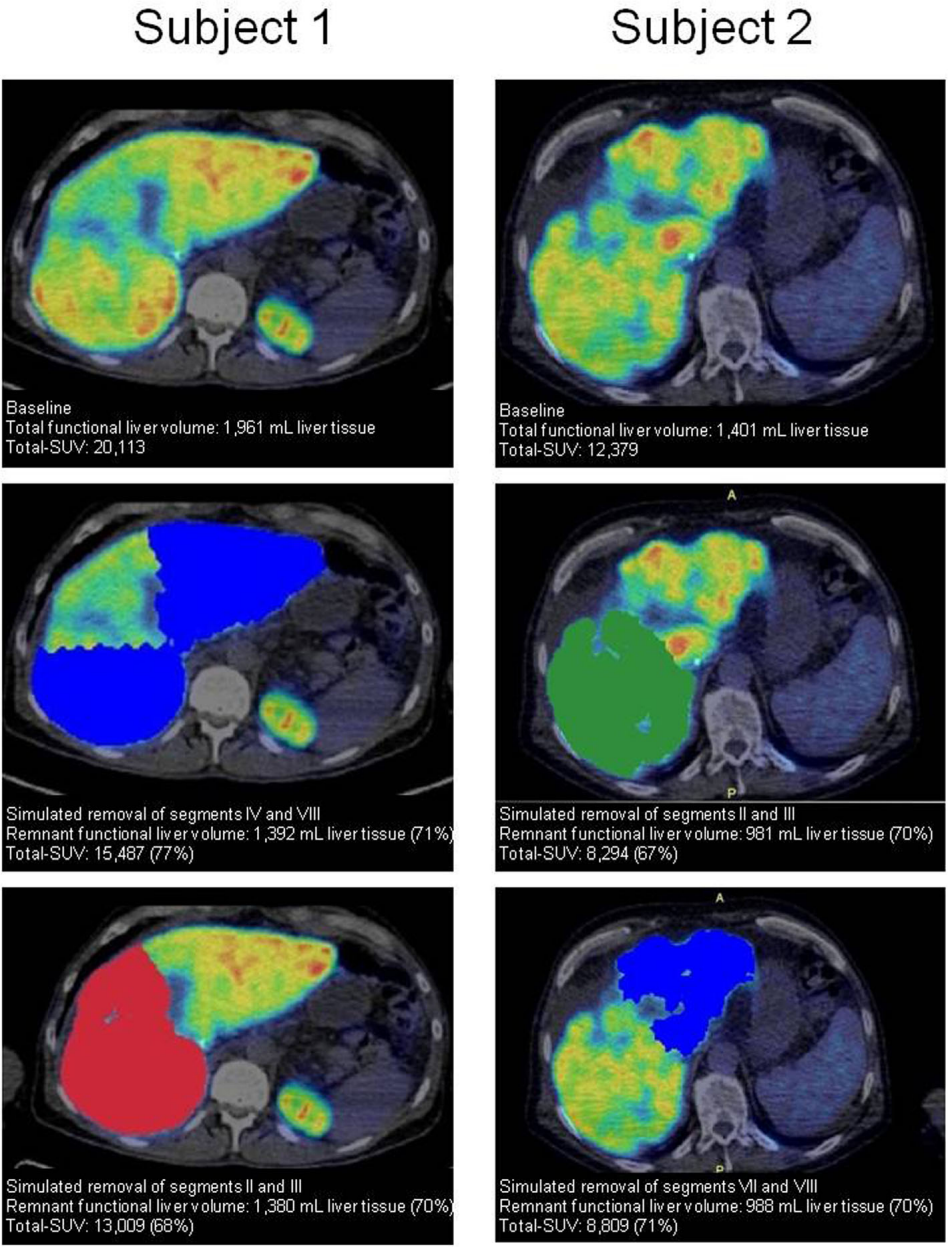
Effect on remnant functional liver volume and total SUV of ^18^F-FDGal by simulated resections of 30% of total functional liver volume in two patients with cirrhosis. The effect is calculated in percentage of baseline values

For patients with cirrhosis, who are considered for partial hepatectomy, a remnant liver volume of 25–90% is recommended [6]. This wide range not only reflects differences in disease severity and comorbidity but also the fact that the anatomical volume does not necessarily reflect liver function in patients with cirrhosis who also have reduced regeneration capacity [18]. The assessment must thus be individual and is typically based on contrast-enhanced CT volumetry, i.e. anatomy and not functional liver volume [6, 19]. The low day-to-day variation of the total functional liver volume and total SUV are important features of ^18^F-FDGal PET/CT as a non-invasive assessment of regional metabolic liver function with particular focus on regional heterogeneity. ^18^F-FDGal PET/CT may therefore be an important supplement to CT for planning individual treatment modality. Based on the findings in the healthy subjects, a total SUV of minimum 6500, equivalent to 25% of the upper limit of the 95% confidence interval for total SUV in healthy subjects, could be proposed as a general threshold for minimum remnant liver function. Figure 4 shows examples of simulated resections of 30% of the total functional liver volume with estimated remnant liver function in two patients with different severity of cirrhosis (subject 1 Child-Pugh class A vs. subject 2 Child-Pugh class B). As shown, removing segments IV and VIII in subject 1 leaves almost 15,500 (80%) of total SUV, a value well above the suggested 6500. On the other hand, removing segments II and III in subject 2 leaves a total SUV of 8294, close to the abovementioned critical level of liver function. These examples illustrate how surgery could be extended in subject 1 but should be carefully considered in subject 2. Because we did not perform CT liver volumetry, the fractional liver volumes in these examples are based on the total functional liver volume but this application of ^18^F-FDGal PET/CT for estimation of remnant liver function of course needs confirmation in a larger dedicated prospective study.

Another promising application of ^18^F-FDGal PET/CT is in planning stereotactic body radiotherapy of liver tumours. We recently showed that functional treatment planning using ^18^F-FDGal PET/CT significantly reduced the radiation dose delivered to the best functioning areas of surrounding liver tissue when treating patients with liver tumours [7].

## Conclusions

The present findings demonstrate that SUV can substitute hepatic systemic clearance, *K*_met_, for functional imaging and quantification of the regional and total hepatic metabolic capacity using ^18^F-FDGal PET/CT. This simplification and the low individual day-to-day variation makes ^18^F-FDGal PET/CT promising for treatment planning of patients with cirrhosis and liver tumours.

## Abbreviations

^18^F–FDGal: 2-^18^F-fluoro-2-deoxy-D-galactose
^99m^Tc: ^99m^Technetium
AIH: Autoimmune hepatitis
Gd-EOB-DTPA: Gadolinium ethoxybenzyl diethylene-triaminepentaacetic acid
HCV: Hepatitis C virus
*K*_met_: Hepatic systemic clearance of ^18^F-FDGal
PBC: Primary biliary cholangitis
ROI: Region of interest
SUV: Standardised uptake value
Total SUV: Total metabolic liver function
VOI: Volume of interest
